# Association between the frequency of treating foreign patients and the cultural competency of Japanese healthcare professionals: a mixed-method study

**DOI:** 10.1186/s41182-025-00844-z

**Published:** 2025-11-24

**Authors:** Yu Par Khin, Sumire Kimura, Seiya Shibata, Nobutoshi Nawa, Takeo Fujiwara

**Affiliations:** 1 Department of Public Health, Institute of Science Tokyo, 1-5-45 Yushima, Bunkyo-ku, Tokyo, Japan; 2 Center for Well-being Research Advancement, Institute of Science Tokyo, 1-5-45 Yushima, Bunkyo-Ku, Tokyo, Japan; 3 Department of Global Health Entrepreneurship,, Institute of Science Tokyo, 1-5-45 Yushima, Bunkyo-ku, Tokyo, Japan

**Keywords:** Cultural competence, Foreign patients, Healthcare professionals, Japan, Mixed-methods

## Abstract

**Background:**

Previous studies have emphasized that interactions with foreign patients were associated with high cultural competence among Japanese healthcare professionals (J-HCPs), with little focus on the individual components of cultural competence. This study examines how frequencies of treating foreign patients are associated with the components of cultural competence among J-HCPs, using a mixed-method design.

**Methods:**

Quantitative data were collected from 1089 J-HCPs via internet survey assessing cultural competence using the Cross-Cultural Competence Instrument for Healthcare Professionals (J-CCCHP), containing subscales, motivation/curiosity, emotion/empathy, attitude, and skill. Associations were stratified by the participation in trainings for treating foreign patients. Qualitative data were further collected from 16 key-informant interviews recruited by snowball sampling.

**Results:**

J-HCPs who treated foreign patients several times a week (n = 203, 18.6%) scored a lower attitude score (coefficient = − 0.67, 95% Confidence Interval [CI] − 1.28, − 0.06), and a higher skill score (coefficient = 1.36, 95% CI 0.43, 2.29) compared to those who treated almost none. Those who treated foreign patients several times a year scored higher in motivation/curiosity, additionally. Qualitative studies explained that rewarding experiences and gaining extensive knowledge in treating foreign patients enhanced J-HCPs’ motivation/curiosity and skill. Stress due to extra workload, language barriers and cultural differences, insufficient resources and the lack of institutional support might lower the attitude of J-HCPs.

**Conclusions:**

Treating foreign patients is associated with high motivation and skill but low attitude scores among J-HCPs, due to systemic challenges. Providing reliable interpretation services, offering practical cultural competence training, and strengthening institutional support may help reduce these challenges.

**Supplementary Information:**

The online version contains supplementary material available at 10.1186/s41182-025-00844-z.

## Background

According to the Lancet Commission on Culture and Health, culture is defined as “the ideas, symbols, and concrete artifacts that sustain conventions and practices, and make them meaningful.”[1]. Culture impacts healthcare in multiple ways, including how individuals perceive health and deal with an illness [2, 3], when they seek healthcare, and which treatment they choose [4]. However, clinical practices often tend to standardize human nature, frequently overlooking the cultural influences on patients’ well-being, especially during encounters with patients from diverse cultural backgrounds [1]. Lack of consideration on cultural differences often leads to miscommunication between patients and healthcare professionals, which in turn contributes to unequal health outcomes for patients from diverse cultural backgrounds [5].

To effectively deliver healthcare services that meet the social, cultural, and linguistic needs of patients, the concept of cultural competence in healthcare was introduced [6]. Several literatures have demonstrated that culturally competent care improved clinical outcomes, such as increased patient satisfaction [7], improved outcomes of diabetic patients [8], and reduced asthma-related hospitalizations [9]. There have been several conceptualizations of cultural competence depending on different disciplines. Still, many accepted that the core components of cultural competence include beliefs, attitudes, knowledge and skills of healthcare professionals to care for patients with diverse cultural backgrounds as proposed by Sue et al. [10]. However, research on clinical cultural competence and its components has not been widely studied in culturally homogeneous settings like Japan.

Japan, a renowned tourist destination that welcomed 36.8 million international visitors in 2024 [11], is also experiencing a steady increase in its migrant population, reaching 3.8 million in the same year [12]. International visitors are individuals who travel to Japan for a short-term stay, usually not exceeding 90 days. Migrants refer to individuals who move to Japan from their home country for the purpose of settlement, work or study and typically staying longer than 90 days [13]. Both can be considered foreign patients in the context of Japanese healthcare, a term that refers to non-Japanese nationals who access medical services in Japan [14].

In response to the increase of foreign patients with diverse cultural backgrounds, the Ministry of Health, Labor and Welfare published a manual for medical institutions on accepting foreign patients [15]. This manual stated the importance of improving healthcare professionals’ knowledge and skills in treating foreign patients and encouraged the implementation of relevant training programs in collaboration with local stakeholders [15]. One such program by Tokyo metropolitan government provides training for healthcare professionals through online video conferencing or freely accessible online videos [16]. A systematic review of cultural competence training outcomes across various countries revealed that such training significantly improves healthcare professionals’ cultural competence, particularly in knowledge and skills [17]. However, limited research has been conducted on the effectiveness of these initiatives in Japan.

Existing literature on the clinical cultural competence in Japan, focused solely on the cultural competence of nurses [18–20]. They have found that the cultural competence level of Japanese nurses was lower than that of nurses from other countries [18] and encounters with foreign patients enhanced their cultural competence [19, 20]. However, there is a lack of literature on the cultural competence of Japanese healthcare professionals (J-HCPs) beyond nurses, as well as factors determining each component of cultural competence. Moreover, little study observes how participation in training programs altered these associations. To address these gaps, this study examines the associations between frequency of treating foreign patients and components of cultural competence among J-HCPs, considering participation in relevant training programs, using an explanatory sequential mixed-methods design.

## Methods

### Quantitative study

#### Sample population

We collected data from J-HCPs of 10 prefectures with significant foreign population (Ibaraki, Saitama, Tokyo, Chiba, Kanagawa, Shizuoka, Aichi, Osaka, Hyogo and Fukuoka prefectures) through a web-based survey using Rakuten Insight [21]. Electronic informed consents were obtained by having them check a consent box prior to participation. Survey was distributed to 8748 panelists who were identified as doctors, dentists or nurses, among them 1100 responded (response rate 12.3%). We excluded those whose workplace city and prefecture were not matched (n = 11, 1.0%), as this can be interpreted as potential misunderstanding during survey completion. The final sample size was 1,089 healthcare professionals.

### Variables

As the outcome, Japanese validated version of cross-cultural competence instrument for healthcare professionals (J-CCCHP) was used as it is applicable to all types of J-HCPs and has acceptable psychometric properties [comparative fit index (CFI) = 0.92 for validity, Cronbach’s alpha = 0.85 for reliability] [20]. It includes 4 subscales adjusted to the context of J-HCPs: (1) motivation/curiosity measuring J-HCPs’ motivation to provide culturally responsive care and their curiosity to engage in and learn from cross-cultural encounters, (2) emotion/empathy measuring J-HCPs’ feelings, emotions and empathy for culturally diverse patients, (3) attitudes measuring J-HCPs’ tolerance and positive orientation towards cultural diversity, and (4) skills measuring J-HCPs’ culturally competent skills in clinical practice [22]. The motivation/curiosity and skills subscales include 7 items each, while the emotion/empathy and attitudes subscales include 5 items each. Each item was rated on a five-point Likert scale. Subscale scores were calculated by summing up the responses of each item, resulting in the range of 7 to 35 for, motivation/curiosity and skills subscales, and 5 to 20 for emotion/empathy and attitudes subscales. Total score was calculated by summing up all subscale scores, and ranges from 24 to 120. All these scores were treated as continuous variables.

For exposure, the frequency of treating foreign patients was assessed using six response categories: almost every day, once or twice a week, once or twice a month, several times in a year, less than several times a year, and none. These responses were grouped into three categories for analysis: several times a week (almost every day and once or twice a week), several times a year (once or twice a month and several times in a year), and almost none (less than several times in a year and none). Covariates included age (categorized as less than 30 years, 30–39 years, 40–49 years, 50–64 years, and more than or equal 65 years), sex (male, female), job type (dentist, doctor and nurse), the type of facility they are working at (university hospital, other hospital, clinics and others), job location (rural and urban), the participation in any form of training for treating foreign patients (yes or no) and the experience of working as a medical professional in a foreign country (yes or no). These covariates were selected based on previous literature on the cultural competency of J-HCPs [18], as they were considered to confound the associations between exposure and outcome.

### Analysis

Multivariable linear regression was conducted to examine the associations between the frequency of treating foreign patients and the total and subscales of the J-CCCHP. Model 1 was adjusted for age, gender, job type, job location, type of facility and experience working in a foreign country. Model 2 was additionally adjusted for the participation in training for treating foreign patients. All variables were complete without missing data. We assume linearity as the mean of each categorical exposure has linear relationships with the outcome [23]. Independence was assumed by plotting the residuals against dummy variables [23]. We conducted independent *t* tests for variables with two categories and one-way ANOVA for variables with more than two categories. Collinearity between variables was assessed using Pearson correlations and variance inflation factors (VIF); all correlation coefficients were below 0.4, indicating weak correlations, and all VIF scores were below 5, suggesting no high multicollinearity. Statistical significance was defined as a *p* value less than 0.05, using a two-sided confidence interval. Given that participation in training for treating foreign patients may enhance the cultural competence of J-HCPs [17], we aimed to determine whether such participations modify the associations between treating foreign patients and cultural competence. To assess this, previous analyses were further stratified by the participation in training. The potential confounders such as age, gender, job type, job location, the type of facility, and the experience of working in a foreign country were adjusted for these stratifications. Stata version 16.0 was used for the analysis.

### Qualitative study

To explain the quantitative findings, we conducted 16 key-informant interviews with J-HCPs recruited through snowball sampling. Eligible participants who were working in tertiary hospitals or clinics in Tokyo and Saitama prefectures and were treating foreign patients several times a week, were recruited through authors’ personal connections. These two prefectures were chosen due to their high concentration of medical facilities that regularly serve foreign patients. We established participant diversity by including a range of J-HCPs, such as doctors, nurses and dentists. There was no overlap with participants from quantitative research. Each interview lasted around 30 min to 1 h. Questions included their experiences and opinions on treating foreign patients, receiving training and support to treat foreign patients and how the frequency of treating foreign patients impacts their cultural competence. YPK and SK conducted the interviews in Japanese. Interviews were continued until the saturation was reached, where no new themes or insights could be obtained from subsequent interviews [24]. This was assessed through qualitative data analysis conducted in parallel with the data collection. The interviews were transcribed verbatim, and thematic analysis was conducted manually. Three researchers (YPK, SK and SS) independently coded the transcripts. In cases of disagreement, fourth author (NN) was consulted to reach consensus. The finalized codes were then organized into overarching themes and subthemes, which were categorized according to the quantitative findings.

### Patient and public involvement

The results of this study will be shared with the public through the website and Twitter account of the corresponding author’s affiliated department. These materials will be tailored for a general audience with plain-language summaries, figures and key messages.

### Ethical consideration

The study was approved by the Medical Ethics Committee, Institute of Science Tokyo (decision no. M2023-383–02). For participants in the online survey, electronic consent was obtained by having them check a consent box prior to participation. For interview participants, written or oral consents were obtained, depending on what was convenient for them.

## Results

Table 1 presents the characteristics of J-HCPs in quantitative study and their total J-CCCHP scores. Among 1,089 J-HCPs, nearly one-third of the participants (32.6%) were 50–64 years. Male and female accounted for 41.9% and 58.1%, respectively. Nurses were the largest percentage (62.4%) followed by doctors (27.1%) and dentists (10.5%). Almost half of them were working at a hospital other than university hospital (47.5%) and 60.2% in urban areas. Only 41 (3.8%) had the experience of working in a foreign country and 119 (10.9%) had participated in training for treating foreign patients. Regarding the frequency of treating foreign patients, 18.6% treated foreign patients several times a week, 47.4% treated several times a year and 34.0% treated almost none in a year. J-HCPs who were aged 65 years or older, male, dentists, working at clinics were significantly associated with lower J-CCCHP total scores. In addition, lower scores were significantly associated with those who had no experience of working in a foreign country and had not participated in training for treating foreign patients (*p* value < 0.05). Supplementary Table 1 describes total and subscales of J-CCCHP across different frequencies of treating foreign patients.

**Table 1:** Characteristics of participants in quantitative study (N = 1,089)

			Total score of J-CCCHP	
		n (%)	Mean (SD)	*p* value*
Age	Less than 30 years	119 (10.9%)	80.2 (11.9)	**0.003**
	30–39 years	206 (18.9%)	80.8 (11.0)	
	40–49 years	314 (28.8%)	82.9 (11.6)	
	50–64 years	355 (32.6%)	79.8 (13.1)	
	More than or equal 65 years	95 (8.7%)	78.4 (13.6)	
Sex	Male	456 (41.9%)	77.7 (12.9)	** < 0.001**
	Female	633 (58.1%)	83.1 (11.3)	
Job type	Dentist	114 (10.5%)	76.1 (14.1)	** < 0.001**
	Doctor	295 (27.1%)	77.1 (12.7)	
	Nurses	680 (62.4%)	83.2 (11.1)	
Type of facility	University hospital	123 (11.3%)	82.2 (11.0)	** < 0.001**
	Other hospital	517 (47.5%)	80.8 (11.8)	
	Clinic	290 (26.6%)	78.7 (14.1)	
	Others	159 (14.6%)	83.6 (10.6)	
Job location	Rural	433 (39.8%)	81.4 (12.0)	0.166
	Urban	656 (60.2%)	80.4 (12.5)	
Experience of working in a foreign country	No	1048 (96.2%)	80.7 (0.38)	0.050
	Yes	41 (3.8%)	84.6 (1.9)	
Participation in training for treating foreign patients	No	970 (89.1%)	80.4 (12.3)	**0.003**
	Yes	119 (10.9%)	84.0 (11.6)	
Frequency of treating foreign patients	Several times a week	203 (18.6%)	80.5 (13.5)	0.488
	Several times a year	516 (47.4%)	81.3 (12.2)	
	Almost none	370 (34.0%)	80.4 (11.7)	

J-*CCCHP* Japanese validated version of cross-cultural competence instrument for healthcare professionals

*n* number, *SD* Standard deviation

^*^p values represent comparisons across all groups., Bold: *p* value < 0.05

Table 2 describes the associations between the frequency of treating foreign patients and the total and subscales of the J-CCCHP. In Model 1, J-HCPs who treated foreign patients several times a year had a significantly higher total score of 2.36 points (95% confidence interval, CI 0.68, 4.04) compared to those who treated almost no foreign patients. This association was still significant in Model 2. Regarding subscales in Model 1, treating foreign patients several times a year was significantly associated with a higher motivation/curiosity score of 1.38 points (95% CI 0.55, 2.22). Treating foreign patients several times a week and several times a year were also significantly associated with higher skill scores of 1.44 points (95% CI 0.51, 2.37) and 1.48 points (95% CI 0.75, 2.21), respectively. However, compared to those who treated almost no foreign patients, these groups significantly demonstrated lower attitude scores of 0.69 points (95% CI − 1.29, − 0.08) and of 0.52 points (95% CI − 1.00, − 0.04), respectively. All these associations were still significant in Model 2 after adjustment for the participation in training.

**Table 2:** Association between the frequency of treating foreign patients and J-CCCHP*

Frequency of treating foreign patients	Total Score		Motivation/Curiosity		Emotion/Empathy		Attitude		Skill	
	Model 1	Model 2	Model 1	Model 2	Model 1	Model 2	Model 1	Model 2	Model 1	Model 2
Several times a week	1.84 (− 0.30, 3.98)	1.66 (− 0.48, 3.80)	0.76 (− 0.30, 1.82)	0.65 (− 0.41, 1.71)	0.33 (− 0.30, 0.95)	0.32 (− 0.30, 0.94)	− **0.69** **(**− **1.29, -0.08)**	− **0.67** **(**− **1.28, **− **0.06)**	**1.44** **(0.51, 2.37)**	**1.36** **(0.43, 2.29)**
Several times a year	**2.36** **(0.68, 4.04)**	**2.24** **(0.56, 3.92)**	**1.38** **(0.55, 2.22)**	**1.32** **(0.48, 2.15)**	0.01 (− 0.47, 0.50)	0.01 (− 0.48, 0.50)	− **0.52** **(**− **1.00, **− **0.04)**	− **0.51** (− **0.99, **− **0.03)**	**1.48** **(0.75, 2.21)**	**1.43** **(0.70, 2.16)**
Almost none	ref	ref	ref	ref	ref	ref	ref	ref	ref	ref

Model 1: Adjusted for age, gender, job type, the type of facility, job location and the experience of working in a foreign country

Model 2: Adjusted for Model 1 + participation in training for treating foreign patients

*J-CCCHP: Japanese validated version of cross-cultural competence instrument for healthcare professionals (Total and subscales), ref: reference, Bold: *p* value < 0.05

Positive values reflect higher cultural competence

Supplementary Table 2 describes the total and subscales of the J-CCCHP, stratified by the participation in training of treating foreign patients.

Table 3 describes the associations between the frequency of treating foreign patients and the total and subscales of J-CCCHP among those who had not participated in training. J-HCPs who treated foreign patients several times a week and several times a year had significantly higher total scores than those who treated almost no foreign patients. Regarding the subscales, treating foreign patients several times a year was significantly associated with a higher motivation/curiosity score of 1.29 points (95% CI 0.42, 2.16). Treating foreign patients several times a week and several times a year were significantly associated with higher skill scores of 1.61 points (95% CI 0.61, 2.61) and 1.58 points (95% CI 0.82, 2.34), respectively. However, these groups demonstrated significantly lower attitude scores of 0.43 points (95% CI − 1.07, − 0.22) and of 0.42 points (95% CI − 0.91, − 0.08), respectively. Supplementary Fig. 1a illustrates these findings for clarification.

**Table 3:** Association between the frequency of treating foreign patients and J-CCCHP* among those who had not participated in training (N = 970)

Frequency of treating foreign patients	Total score	Motivation/Curiosity	Emotion/Empathy	Attitude	Skill
Several times a week	**2.27 (0.00, 4.54)**	0.71 (− 0.43, 1.84)	0.38 (− 0.27, 1.04)	− **0.43 (**− **1.07, 0.22)**	**1.61 (0.61, 2.61)**
Several times a year	**2.44 (0.70, 4.18)**	**1.29 (0.42, 2.16)**	− 0.01 (− 0.51, 0.49)	− **0.42 (**− **0.91, 0.08)**	**1.58 (0.82, 2.34)**
Almost none	ref	ref	ref	ref	ref

Adjusted for age, gender, job type, the type of facility, job location and the experience of working in a foreign country

*J-CCCHP: Japanese validated version of cross-cultural competence instrument for healthcare professionals (Total and subscales), ref: reference, Bold: *p* value < 0.05

Positive values reflect higher cultural competence

Table 4 describes the associations among J-HCPs who had participated in training. Compared to those who treated almost no foreign patients, those who treated foreign patients several times a week and several times a year had significantly lower attitude scores of − 2.86 points (95% CI − 5.08, − 0.65) and -2.31 points (95% CI − 4.27, − 0.34), respectively. There were no significant associations for other subscales and the total score. Supplementary Fig. 1b illustrates these findings for clarification.

**Table 4:** Association between the frequency of treating foreign patients and J-CCCHP* among those who had participated in training (N = 119**)

Frequency of treating foreign patients	Total Score	Motivation/Curiosity	Emotion/empathy	Attitude	Skill
Several times a week	− 2.43 (− 10.15, 5.30)	1.35 (− 2.43, 5.13)	− 0.34 (− 2.68, 2.00)	− **2.86 (**− **5.08, **− **0.65)**	− 0.57 (− 3.64, 2.50)
Several times a year	− 1.71 (− 8.57, 5.15)	1.82 (− 1.54, 5.18)	− 0.34 (− 2.42, 1.74)	− **2.31 (**− **4.27, **− **0.34)**	− 0.89 (− 3.61, 1.84)
Almost none	ref	ref	ref	ref	ref

Adjusted for age, gender, job type, the type of facility, job location and the experience of working in a foreign country

*J-CCCHP: Japanese validated version of cross-cultural competence instrument for healthcare professionals (Total and subscales), ref: reference, Bold: *p* value < 0.05

Positive values reflect higher cultural competence

^**^Due to the small sample size, confidence intervals are wider with less precise estimates

To explain quantitative findings of higher frequencies of treating foreign patients were associated with higher motivation/curiosity and skill scores and lower attitude scores, we conducted qualitative in-depth interviews with J-HCPs who were treating foreign patients several times a week. Supplementary Table 3 presents the characteristics of J-HCPs in qualitative study. They included 7 doctors, 1 dentist and 8 nurses, with ages ranging from 28 to 77 years. All doctors and the dentist were male, and all nurses were female. Four of them worked at clinics, where the rest worked at hospitals, and five reported prior experience of working in a foreign country.

Table 5 presents qualitative interpretations of quantitative findings according to J-HCPs. High motivation/curiosity scores among J-HCPs who treated foreign patients frequently were explained by rewarding experiences. High skill scores were explained by gaining extensive knowledge in treating foreign patients. Lower attitude scores were explained by the stress they experienced due to extra workload, language barriers and cultural differences, with underlying insufficient resources and the lack of institutional support.

**Table 5:** Qualitative interpretations of quantitative findings

Quantitative themes	Subthemes	Brief explanation
High motivation/curiosity scores	Rewarding experiences	J-HCPs* felt rewarding to help foreign patients
High skill scores	Gaining extensive knowledge in treating foreign patients	J-HCPs* gained extensive knowledge about foreign patients
Low attitude scores	Stress due to extra workload	J-HCPs* felt stressed to care for foreign patients due to extra documentations
	Stress due to language barriers and cultural differences	J-HCPs* felt stressed due to language barriers and when foreign patients insisted on following their own cultural practices
	Insufficient resources	J-HCPs* felt challenges due to insufficient resources such as insufficient interpreters
	Lack of institutional support	Current healthcare system could not keep in pace with increasing foreign patients

^*^J-HCPs: Japanese healthcare professionals

### Qualitative findings

#### High motivation/curiosity scores

Since providing care for foreign patients was difficult for both foreign patients and J-HCPs, some found it rewarding to be able to help foreign patients, which could further motivate them. *“When patients and hospital staff express their gratitude for our support, it makes my job very rewarding.”* (D03).

### High skill scores

Throughout years of experience treating foreign patients, J-HCPs gained extensive knowledge about foreign patients. *“We interact (with foreign patients) almost every day, so I think we can get a sense of how to deal with foreign patients.”* (N07).

### Low attitude scores

J-HCPs noted that their positive attitude towards foreign patients can be affected because of the stress associated with extra workload required to care for foreign patients. Tasks such as filling insurance forms and contacting oversea institutions in English were especially burdensome*.* One noted, “*To be honest, we don't get anything for it, so it does put a bit of a burden on us.”* (D05).

Language barriers and cultural differences were also major sources of stress. *“It is difficult for us to understand (foreign patients), which makes us feel sorry*.” (N06) Tensions increased when foreign patients insisted on following their own cultural practices during healthcare.* “Sometimes they (foreign patients) try to push through their (cultural) demands… that might have a slightly negative impact on Japanese people.”* (D07).

These challenges stemmed from insufficient resources, such as limited number of interpreters. *“At our current hospital, there are only about 10 English interpreters, so it's not always clear whether one will be available…”* (N08) Many J-HCPs also emphasized that current healthcare system could not keep in pace with the increasing numbers of foreign patients, which was an urgent need. *“Measures are urgently needed, and reforms need to be led by the government.”* (D03).

Supplementary Table 4 describes themes and subthemes from the recommendations of J-HCPs. They recommended training that reflects real life experiences, language training and other recommendations, such as developing manuals to support foreign patients and the involvement of third-party assistance.

### Recommended training

Regarding the training programs of J-HCPs for treating foreign patients, even though some participants heard or attended them, they felt the applicability of these trainings on their clinical practice is limited. J-HCPs recommended that training programs should better reflect real-life clinical situations. *“It would be great if there was a training session like, role-playing…”* (N01) Some also expressed the need for English medical education for J-HCPs during their university years.* “I wish we had something like English classes.”* (N03).

### Other recommendations

Some J-HCPs preferred having manuals to guide them through difficult situations while treating foreign patients. *“I think it would be nice to have some kind of manual for dealing with foreigners.”* (N07).

Many J-HCPs strongly recommended the establishment of support groups, either internal (within the healthcare facility) or external (through non-governmental organizations (NGOs)).* “If I had to deal with (foreign) patients at a hospital that does not have an international medical department, it would be extremely difficult…”* (D04)* “They (NGOs) are knowledgeable about things like visa status or financial issues. So, I was able to get support in those areas.”* (D06).

## Discussion

This study examined the association between frequencies of treating foreign patients and cultural competence among J-HCPs. Despite modest score differences (1–2 points), effect size estimates (Cohen’s f^2^ = 0.03–0.25) indicate small to medium associations, supporting the practical relevance of the findings [25]. Treating foreign patients was associated with high total score of cultural competence which is similar to previous studies [19, 20]. Particularly, treating foreign patients frequently was associated with higher motivation/curiosity and skill scores due to rewarding experiences and extensive knowledge gained. Japanese population has been homogenous, and Japanese healthcare is particularly tailored for the Japanese population [26]. The increase in foreign patients, as tourists or migrants, is relatively recent. Only a certain proportion of J-HCPs working in urban areas have the exposure with foreign patients [27], and these J-HCPs understand how to deal with foreign patients through their experience. Moreover, communication barriers enhanced difficult experiences for both foreign patients and J-HCPs, and that overcoming these situations successfully is rewarding [28], enhancing the motivation and curiosity of J-HCPs to treat foreign patients.

This study found that frequent exposure to foreign patients was inversely associated with the attitude subscale of cultural competence of J-HCPs probably due to stress, limited resources and systemic constraints. Resource availability for J-HCPs highly varies across healthcare facilities [29]. Even in institutions that provided interpreters and translation tools, several limitations were identified, including the insufficiency of qualified interpreters [30] and the accuracy of translation technologies [31, 32]. Moreover, developing these systems largely relies on individual facility resources, with limited support from the national government [33, 34]. Consequently, J-HCPs reported feeling helpless and stressed when caring for foreign patients [35, 36]. To address this, qualitative findings recommended relying on non-governmental organizations, which play a crucial role in supporting foreign patients [34]. They also emphasized the need for clear guideline and transparency, particularly when caring for foreign patients without health insurance or documentations, which is also supported by the previous literature conducted across 16 European countries [37].

Although cultural competence training was cited as effective by previous literature [17, 38], this study found that frequently treating foreign patients was associated with lower attitude scores even among J-HCPs who had training participation. This might be due to the limited practicality and relevance of the training as the qualitative study suggested. For cultural competence training to be effective, cultural competence training must be tailored to the specific needs of its recipients [39]. In Japan, where the healthcare system is primarily prepared for the Japanese population [26], it is essential that J-HCPs receive training that prepares them to care for patients with diverse cultural backgrounds and expectations while ensuring that the training content is directly applicable for their clinical practices.

This study has several limitations. First, since the study was conducted via an internet-based survey, only J-HCPs registered at the internet survey company were eligible to participate. As a result, the sample may have been skewed towards individuals who were more likely to be women, older, and residing in larger urban areas [40], limiting the generalizability of the findings. Moreover, the number of J-HCPs who participated in the quantitative study represents less than 1% of the total J-HCP population in the selected prefectures based on the latest available data from the Ministry of Health, Labor and Welfare [41]. As such, the sample may not be fully representative of the whole population of J-HCPs in each prefecture. Second, regarding the subscales of cultural competence, there are limitations to interpret each subscale separately as their conceptual context can overlap, particularly between attitude vs. emotion/empathy. This overlap may make it difficult to attribute observed effect to the single domain of cultural competence. Third, there is a possible information bias due to the self-reported nature of the survey. Fourth, reverse causality is also possible due to the cross-sectional design of the study. Fifth, the recruitment via personal networks and snowball sampling in the qualitative study may limit the transferability of the findings to a broader context. We also could not conduct the member checking for the qualitative research due to practical constraints to re-engage busy J-HCPs after the initial data collection. However, we have achieved credibility of the data through the triangulation of quantitative and qualitative data. Finally, the sample size of the J-HCPs who had participated in training is relatively small (n = 119) and details of the training programs including the content, duration and providers were not available, limiting the interpretation of training participation.

However, this study applied mixed-methods approach to provide robust evidence on the association between the frequency of treating foreign patients and the cultural competence of different types of J-HCPs, using the J-CCCHP [20]. To enhance the cultural competence of J-HCPs, reliable interpretation services, and practice-based cultural competence training are essential. In addition, clear guidelines, government support and assistance from non-governmental organizations are also necessary to create supportive environment for J-HCPs caring for foreign patients.

## Conclusions

Although treating foreign patients was associated with higher scores on the motivation/curiosity and skill subscales of cultural competence among J-HCPs, it was also associated with lower scores on the attitude subscale. The overall improvement of cultural competence can be achieved through targeted trainings and institutional support.

## Supplementary Information

Supplementary file 1.

## Data Availability

Not available.

## Abbreviations

J-HCPs: Japanese healthcare professionals
J-CCCHP: Japanese validated version of cross-cultural competence instrument for healthcare professionals
