# Supplementary material for: Association between the frequency of treating foreign patients and the cultural competency of Japanese healthcare professionals: a mixed-method study
Source: Trop Med Health. 2025 Nov 24;53:171. doi: 10.1186/s41182-025-00844-z (PMC12642154; doi:10.1186/s41182-025-00844-z)
Supplement: Supplementary file 1 [file 41182_2025_844_MOESM1_ESM.docx]

Supplementary Table 1: Total and subscales of J-CCCHP among participants

|  |  |  | Total Score | | Motivation/Curiosity | | Emotion/Empathy | | Attitude | | Skill | |
| --- | --- | --- | --- | --- | --- | --- | --- | --- | --- | --- | --- | --- |
|  |  | n (%) | Mean (SD) | p-value* | Mean (SD) | p-value* | Mean (SD) | p-value* | Mean (SD) | p-value* | Mean (SD) | p-value* |
| Frequency of treating foreign patients | Several times a week | 203 (18.6%) | 80.5 (13.5) | 0.488 | 24.5 (6.4) | 0.083 | 15.1 (4.1) | 0.397 | 13.8 (3.6) | **0.002** | 27.0 (5.3) | **0.039** |
|  | Several times a year | 516 (47.4%) | 81.3 (12.2) |  | 25.2 (5.8) |  | 14.5 (3.4) |  | 14.2 (3.4) |  | 27.1 (5.0) |  |
|  | Almost none | 370 (34.0%) | 80.4 (11.7) |  | 24.3 (6.1) |  | 15.0 (3.1) |  | 14.8 (3.3) |  | 26.3 (5.5) |  |

J-CCCHP: Japanese validated version of cross-cultural competence instrument for healthcare professionals

SD: Standard deviation

*p-values represent comparisons across all groups., Bold: p-value<0.05

Supplementary Table 2: Total and subscales of J-CCCHP, stratified by participating in any form of training for treating foreign patients

| Frequency of treating foreign patients | Total Score | | Motivation/Curiosity | | Emotion/Empathy | | Attitude | | Skill | |  |
| --- | --- | --- | --- | --- | --- | --- | --- | --- | --- | --- | --- |
|  | Training (No) | Training (Yes) | Training (No) | Training (Yes) | Training (No) | Training (Yes) | Training (No) | Training (Yes) | Training (No) | Training (Yes) |  |
| Several times a week | 80.0 (13.7) | 82.7 (12.2) | 24.0 (6.5) | 26.8 (5.6) | 15.2 (3.9) | 14.7 (4.9) | **14.0 (3.6)** | **12.8 (3.6)** | **26.8 (5.3)** | 28.4 (5.0) |  |
| Several times a year | 80.9 (12.2) | 84.0 (12.3) | 24.9 (5.8) | 27.0 (5.9) | 14.8 (3.4) | 14.9 (3.1) | **14.2 (3.3)** | **14.1 (3.6)** | **27.0 (5.1)** | 28.0 (4.7) |  |
| Almost none | 80.0 (11.8) | 86.1 (8.2) | 24.2 (6.2) | 25.9 (5.0) | 14.9 (3.1) | 15.5 (2.8) | **14.8 (3.3)** | **15.5 (3.0)** | **26.1 (5.6)** | 29.3 (3.5) |  |
| Bold: p-value<0.05 | | | | | | | | | | | |

J-CCCHP: Japanese validated version of cross-cultural competence instrument for healthcare professionals


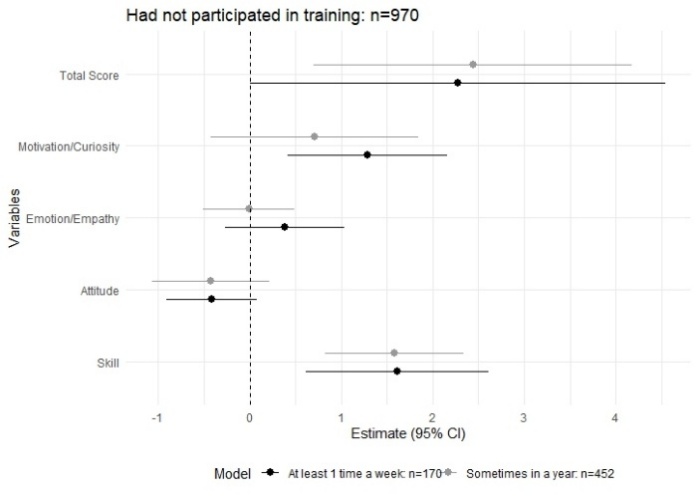

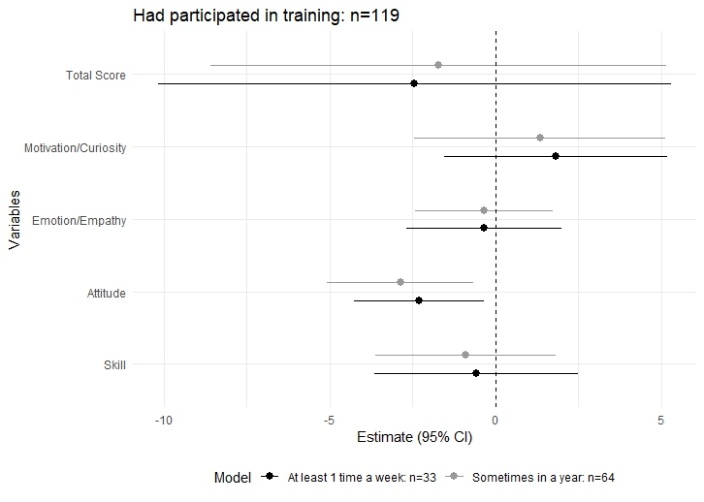


Figure 1 (b)

Supplementary Figure 1: Association between the frequency of treating foreign patients and the total and subscales of the J-CCCHP (a) among those who had not participated in training (b) among those who had participated in training (reference: Frequency of treating foreign patients= Almost none), J-CCCHP: Japanese validated version of cross-cultural competence instrument for healthcare professionals

Figure 1 (a)

Supplementary Table 3: Characteristics of participants in qualitative study (N=16)

|  | **Age** | **Sex** | **Job type** | **Type of facility** | **Experience of working in a foreign country** |
| --- | --- | --- | --- | --- | --- |
| D01 | 33 | Male | Doctor | University hospital | No |
| D02 | 34 | Male | Doctor | University hospital | No |
| D03 | 45 | Male | Doctor | University hospital | Yes |
| D04 | 41 | Male | Doctor | University hospital | No |
| D05 | 29 | Male | Doctor | University hospital | No |
| D06 | 33 | Male | Doctor | Other hospital | No |
| D07 | 28 | Male | Doctor | Other hospital | No |
| Dt1 | 77 | Male | Dentist | Clinic | Yes |
| N01 | 37 | Female | Nurse | Clinic | No |
| N02 | 27 | Female | Nurse | Other hospital | No |
| N03 | 29 | Female | Nurse | Clinic | No |
| N04 | 37 | Female | Nurse | Other hospital | Yes |
| N05 | 32 | Female | Nurse | Clinic | No |
| N06 | 35 | Female | Nurse | Other hospital | No |
| N07 | 38 | Female | Nurse | Other hospital | Yes |
| N08 | 29 | Female | Nurse | Other hospital | Yes |

Supplementary Table 4: Recommendations of Japanese healthcare professionals

| **Themes** | **Subthemes** |
| --- | --- |
| Recommended training | Trainings that reflect real life experiences |
|  | Language trainings for healthcare professionals |
| Other recommendations | Developing manuals to support foreign patients |
|  | Involvement of third-party assistance |
